# Driving Digital Transformation During a Pandemic: Case Study of Virtual Collaboration in a German Hospital

**DOI:** 10.2196/25183

**Published:** 2021-02-01

**Authors:** Nicholas R J Frick, Henriette L Möllmann, Milad Mirbabaie, Stefan Stieglitz

**Affiliations:** 1 Department of IT-Strategy MAINGAU Energie GmbH Obertshausen Germany; 2 Department of Oral-, Maxillo- and Plastic Facial Surgery Heinrich-Heine-University Duesseldorf Duesseldorf Germany; 3 Department of Business Studies and Economics University of Bremen Bremen Germany; 4 Professional Communication in Electronic Media/Social Media University of Duisburg-Essen Duisburg Germany

**Keywords:** digital transformation, virtual collaboration, digital health, health care, COVID-19, pandemic, hospital, collaboration, virtual heath, crisis, case study

## Abstract

**Background:**

The COVID-19 pandemic has not only changed the private lives of millions of people but has significantly affected the collaboration of medical specialists throughout health care systems worldwide. Hospitals are making changes to their regular operations to slow the spread of SARS-CoV-2 while ensuring the treatment of emergency patients. These substantial changes affect the typical work setting of clinicians and require the implementation of organizational arrangements.

**Objective:**

In this study, we aim to increase our understanding of how digital transformation drives virtual collaboration among clinicians in hospitals in times of crisis, such as the COVID-19 pandemic.

**Methods:**

We present the lessons learned from an exploratory case study in which we observed the introduction of an information technology (IT) system for enhancing collaboration among clinicians in a German hospital. The results are based on 16 semistructured interviews with physicians from various departments and disciplines; the interviews were generalized to better understand and interpret the meaning of the statements.

**Results:**

Three key lessons and recommendations explain how digital transformation ensures goal-driven collaboration among clinicians. First, we found that implementing a disruptive change requires alignment of the mindsets of the stakeholders. Second, IT-enabled collaboration presupposes behavioral rules that must be followed. Third, transforming antiquated processes demands a suitable technological infrastructure.

**Conclusions:**

Digital transformation is being driven by the COVID-19 pandemic. However, the rapid introduction of IT-enabled collaboration reveals grievances concerning the digital dissemination of medical information along the patient treatment path. To avoid being caught unprepared by future crises, digital transformation must be further driven to ensure collaboration, and the diagnostic and therapeutic process must be opened to disruptive strategies.

## Introduction

The impact of COVID-19, the disease caused by SARS-CoV-2, has not only changed our lives and interactions as human beings but has had substantial consequences for health care systems worldwide [1]. Above all, hospitals must slow the spread of SARS-CoV-2 and further ensure a certain level of daily routine to maintain regular care and treatment of emergency patients. In addition to changes to clinical routines [2,3], this process involves adjustments in the way that clinicians work together.

Collaboration among hospital employees, particularly between physicians and interconnected medical departments, is essential for the overall diagnosis and treatment process [4-7]. Because the health care system is interdisciplinary in nature, goal-driven collaboration enhances the performance of services, quality of care, and patient outcomes [8-11]. Collaboration in hospitals can be defined as a professional alliance between health care specialists from multiple medical disciplines with diverse backgrounds and varying expertise, who jointly provide benefits for patients [12,13]. Although collaboration is considered to be a top priority in hospitals [14], some common issues are frequently experienced by clinicians [15]. Insufficient communication, including improper exchange of medical information, is a prevalent concern [16]. Clinicians manage their tasks in isolation with a lack of mutual understanding, which prevents the sharing of knowledge across departmental boundaries and inhibits collaboration between disciplines [17,18]. Furthermore, hospitals operate in complex clinical infrastructures, with a wide range of information systems containing different medical information; these systems are disconnected from each other and operated by multiple professionals [19,20]. Consistent presentation of medical data is lacking, and the retrieval of information stored across diversified systems is a time-consuming procedure [21,22].

In addition to existing collaborative challenges in hospitals [15], clinicians are being confronted with issues emerging from the COVID-19 pandemic [23]. Medical workers must not only examine individuals who are infected with the novel virus but must also respond to normal emergencies and patient cases. The required response involves substantial dedication and demands the implementation of diverse organizational arrangements as well as safety precautions [24]. Changes in surgery include decreasing the number of elective procedures to gain more capacity for emergency interventions [25]. Staff caring for patients with COVID-19 are separated from those caring for other patients, and postoperative visits are suspended and replaced by telephone calls to prevent in-hospital spread [3]. Furthermore, clinics are divided into multiple teams to prevent quarantine of an entire department, thus providing patient care in the case of infection. The underlying objective of reducing interpersonal communication compensates for any shortfall and maintains clinical operations. However, due to these necessary adjustments, collaboration in hospitals has changed significantly and has rapidly shifted toward virtual environments. Clinicians have started exchanging information using social media or instant messaging, and meetings are increasingly being conducted using applicable and available technology [3,26].

The integration of technologies into existing processes is a key component of the digital transformation [27,28] that improves cross-functional collaboration and coordination among individuals [29,30]. Digital transformation is defined as “a process that aims to improve an entity by triggering significant changes to its properties through combinations of information, computing, communication, and connectivity technologies” [30]. Therefore, digital transformation addresses changes associated with the introduction of new information technology (IT) in current organizational structures [31,32]. Because digital transformation is concerned with improving collaboration between individuals [30], it is closely linked to the computer-supported cooperative work (CSCW) research stream. CSCW determines how technology can increase group communication and work efforts [33]; thus, it is a vital component for understanding the dynamics of digital transformation [34] and associated changes of collaboration in practice [30].

In health care, digital transformation deliberately seeks to answer the question of how the quality of care and its related services can be improved with technology, as both rely on accurate, relevant, integrated and quickly accessible information [35,36]. For example, recent research examined how processes and digital infrastructure must be aligned to drive IT-enabled innovations [37] and whether advanced technologies, such as the Internet of Things (IoT) and artificial intelligence (AI) [38,39], can be integrated to empower caregivers to make evidence-based clinical decisions [28]. Extant research is further concerned with the potential of health information technology (HIT) to improve patient outcomes while reducing costs [40]. One example of HIT that is fundamentally transforming health services is electronic health records (EHRs), which digitally capture patients’ retrospective, concurrent, and prospective information to guide medical treatment [41]. In this realm, scholars are exploring how technology is creating smart hospitals [42], that is, clinical environments with optimized and automated processes based on technological advancements and intelligent facilities to introduce new capabilities and provide an ideal surrounding for patients [42-44].

However, the increasing application of technology also serves as a basis for enhancing information exchange and collaboration in hospitals [15,45-47]. Collaborative visioning promotes joint decision-making and helps to overcome related issues frequently experienced by clinicians [48]. Communication is improved as information is presented transparently for involved experts. For example, central platforms coordinate the diffusion of reports and information exchange among participants [29,49]. The free transfer of medical information across departmental boundaries further establishes a closer alignment with collaborators from other departments, which dissolves silo thinking [50,51]. In addition, the introduction of standards simplifies complex clinical infrastructures and contributes to a feasible exchange of medical data between detached HIT systems; it also assists clinicians in gathering information more quickly [28].

In summary, the technological capacity for enhancing information exchange and collaboration focusing on patient outcomes is widely available [45-47]. Digital transformation and associated technologies are essential to facilitate the exchange of information among clinicians across organizational boundaries, as new forms of collaborative work drive operational performance [15,30,31]. However, in their complex clinical environments, hospitals traditionally struggle to adopt new technologies [19,20], especially when aligning IT-enabled transformations with existing infrastructure [52]. Agarwal et al [17] stated that “an IT-enabled transformation of health care is just beginning, and it cannot happen too fast.” However, the present situation of the COVID-19 pandemic is confronting hospitals with the urgent need to identify valid solutions while maintaining a sufficient level of collaboration and complying with the legal requirements of social distancing. Radical actions and restrictions are unavoidable for hospitals; however, they involve profound implications. Habitual collaboration is altered instantly, with no or only partial consideration of possible influencing factors such as available resources or security and data protection. Furthermore, the entire process of integrating technologies is likely to be faster during a crisis than under normal conditions, as people are conducting sense-making on a different level [53,54]. Disruptive technologies that are being applied to combat the COVID-19 pandemic are replacing traditional communication in health care teams [55] and are fundamental for sustained collaboration in hospitals. The ongoing pandemic is therefore creating a pressing need to rethink collaboration between clinicians; however, research on how digital transformation advocates collaboration in hospitals during a crisis is still lacking. We argue that this topic is of great interest to researchers and practitioners because virtual collaboration in hospitals will increase even further in the near future and persist for a long time. Thus, our research is guided by the following question: *How is digital transformation driving collaboration among clinicians in a time of crisis?*

We report the unique findings of an exploratory case study of a German hospital, in which we observed the introduction of an IT system to enhance collaboration among physicians during the COVID-19 pandemic. Our results are based and on 16 generalized semistructured interviews with physicians from various departments. We explain how clinicians worked together before the ongoing crisis, what has changed as a result of the pandemic, how the introduction of an IT system ensured effective collaboration, and how the digital transformation should continue to transform hospital operations.

We aim to increase our understanding of how digital transformation can improve collaboration among clinicians in the subject hospital and other hospitals. Moreover, it will demonstrate how the proposed IT improvements can ensure accurate, safe, and effective patient care not just for future crises but for daily operations. Researchers will be able to use our key lessons to understand the difference between a regular installation and one made out of necessity, along with their advantages and disadvantages. Practitioners will be able to understand how our recommendations help to ensure goal-driven collaboration and how hospitals can benefit from them. We hope to guide decision makers who want to introduce IT to improve collaboration between clinicians and stimulate additional research in this important field by expanding the body of knowledge.

## Methods

The German health care system is a highly developed sector in which the health of the population and life expectancy has continuously risen [56]. In the fight against SARS-CoV-2, wide-ranging countermeasures were backed by the German government and introduced at an early stage. A crisis taskforce was convened on February 27, 2020, and the first nationwide restrictions on public access were adopted on March 22 [57]. There is no evidence that the system was overburdened, as only 60% of the total of 33,051 intensive care beds were in use [58]. However, the new restrictions undoubtedly influenced hospitals and the ways in which their employees interacted with each other. To determine how collaboration between clinicians changed during the COVID-19 pandemic, we observed the introduction of an IT system at a large German hospital during the period from March 24 to April 24, 2020.

The hospital decided to introduce and provide Microsoft Teams (Microsoft Corporation) throughout the clinic. It was necessary to maintain collaboration between physicians, including those who were working from home, during the pandemic. Furthermore, restricted personal contact was needed to slow the spread of the virus. Microsoft Teams is a collaboration platform with features such as chatting, video calls, and file sharing. It can be used by many devices, such as personal computers, notebooks, tablets, and smartphones [59]. In response to the COVID-19 outbreak, Microsoft decided to offer the Teams software for free to help individuals stay connected [60].

Additionally, several organizational changes were initiated by the hospital. The departments were split into two different teams and worked in a weekly rotation to ensure that if one team was contaminated with the virus, the second team would be able to continue working and providing appropriate patient care. Furthermore, all elective procedures were reduced to a minimum, and only urgent surgeries were performed. However, the most crucial action was the minimization of any face-to-face exchanges between clinicians.

**Figure 1:**
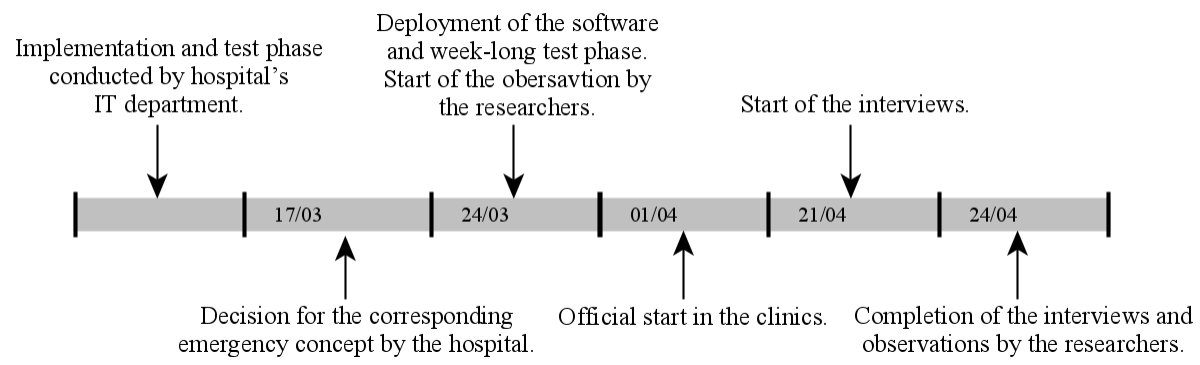
Process of the introduction of Microsoft Teams in the hospital.

As presented in Figure 1, the case study preceded the implementation and test phase conducted by the hospital’s IT department. The decision to implement the corresponding emergency concept, including the rollout of Microsoft Teams, was made on March 17 by the hospital management. On March 24, the software was installed on the employees’ computers. Installation was followed by a week-long test phase during which additional hardware, such as cameras, microphones, and speakers, was installed. Because Microsoft Teams had not been used in the clinic before, clinicians had little or no knowledge of how to use the system. We provided advice and tips exclusively remotely, as visits to the hospital were forbidden to prevent the virus from infecting other patients and spreading. Finally, the first formal meeting and the official start of our support took place on April 1.

After the new system was introduced, we conducted 16 semistructured interviews with physicians. The interviews were designed to provide “questioning guided by identified themes in a consistent and systematic manner” [61]. We developed a guide containing relevant questions in advance. Our goal was to make the guidelines as comprehensive as possible because physicians are typically available for a limited time, especially during a crisis. The guide was divided into chapters, with initial questions and subsequent follow-up questions (see Table 1).

Because we were interested in the meaning of the experts’ substantive statements and not in their linguistic habitus or expressions, which are not necessary for understanding the context, nor in their physical gestures or facial expressions, we decided to paraphrase the interviews instead of conducting verbatim transcriptions. The analysis of the interviews was based on the recommendations in the qualitative assessment of content analysis by Schilling [28]. Paraphrasing the data reduced the volume by removing unnecessary words to form short, concise sentences. We listened carefully to the interview recordings and paraphrased the content of the physicians’ statements. Next, we generalized and reduced the content to better understand and interpret the meaning of the statements [62]. The subsequent categorization was guided by the recommendations of Mayring [29] for an organization of data derived from the material itself. This inductive category formation approach described the data without predefined criteria, leading to an unbiased “understanding of the material in terms of the material” [63].

The physicians were aged between 25 and 42 years (mean 32.2 years, SD 4.4), with 9 female and 7 male experts, and their tenures ranged from 0.5-17 years (mean 4.3 years, SD 4.4). Of the participants, 3 were senior physicians and 13 were resident physicians from 3 different clinics. The interviews were conducted using Microsoft Teams, with which the physicians were by then familiar. The interviews lasted between 9:04 and 28:25 minutes and were recorded, analyzed, and deleted once the evaluation was finished to protect the privacy of the participants. An overview of our sample is outlined in Table 2. The results yielded novel insights on how physicians collaborate with the assistance of technology during a pandemic. They broadened our view on collaboration among physicians in hospitals.

**Table 1:** Interview guideline.

Chapter	Content or questions	Follow-up questions
Before the interview	Introduction and summary of the purpose of this research, including an explanation of the interviewee’s rights and verbal consent for the interview to be recorded	None
Demographic data	1. What is your name and how old are you? 2. What is your current position at the hospital? 3. What tasks does your current position involve?	None
The conditions before the crisis/pandemic	4. How was the collaboration with each other (with your colleagues) before the crisis/pandemic?	a. With whom did you primarily collaborate? b. How did you collaborate? c. Which IT^a^ systems did you use for this purpose?
	5. Where have been problems in the collaboration?	None
The conditions during the crisis/pandemic	6. What was the goal of introducing Microsoft Teams in your hospital or clinic during the crisis/pandemic?	None
	7. How has the introduction changed collaboration during the crisis/pandemic?	a. With whom do you collaborate? b. How has your collaboration behavior changed? c. What types of devices do you use for collaborating? d. Have your tasks or responsibilities changed? e. Has your culture within the clinic changed? Do you treat each other differently?
	8. What advantages have resulted from the introduction in your collaboration?	a. Benefits for the team and/or individuals? b. Benefits for other hospitals employees? c. Benefits for patients?
	9. What disadvantages have arisen from the introduction in your collaboration?	a. Surveillance? b. Data protection? c. Ethical issues or legal questions?
	10. What challenges have been identified during the introduction?	a. Technical problems? b. Acceptance, handling, resistance?
The conditions after the crisis/pandemic	11. Would you like to receive further technical support for collaboration?	a. Further use of Microsoft Teams?
	12. In which other areas would you like to get more support?	a. Artificial intelligence? b. Intelligent systems?
	13. What recommendations would you give other hospitals or their staff to improve collaboration in their clinic?	None
After the interview	Conclusion of the interview and the possibility for the expert to ask further questions or give closing remarks	None

^a^IT: information technology.

**Table 2:** Sample overview of the expert interviews.

Number	Age	Gender	Tenure (years)	Position	Clinic	Length (minutes:seconds)
E1	31	Female	1.5	Resident physician	Craniomaxillofacial surgery	28:25
E2	33	Female	0.5	Resident physician	Craniomaxillofacial surgery	19:21
E3	42	Male	17	Senior physician	Craniomaxillofacial surgery	10:05
E4	32	Male	5	Resident physician	Craniomaxillofacial surgery	17:31
E5	26	Male	0.75	Resident physician	Orthopedics and trauma surgery	12:36
E6	34	Female	6	Resident physician	Craniomaxillofacial surgery	09:04
E7	25	Female	0.3	Resident physician	Otorhinolaryngology	10:57
E8	33	Female	1	Resident physician	Otorhinolaryngology	09:34
E9	29	Male	2	Resident physician	Orthopedics and trauma surgery	14:41
E10	28	Female	2.5	Resident physician	Orthopedics and trauma surgery	14:41
E11	37	Male	7	Senior physician	Craniomaxillofacial surgery	16:33
E12	38	Female	11	Senior physician	Craniomaxillofacial surgery	14:40
E13	29	Female	2.5	Resident physician	Orthopedics and trauma surgery	14:00
E14	32	Female	3.5	Resident physician	Otorhinolaryngology	13:38
E15	31	Male	3.5	Resident physician	Otorhinolaryngology	22:13
E16	36	Male	4	Resident physician	Orthopedics and trauma surgery	15:17

## Results

This section describes the level of collaboration between clinicians before the crisis, what has changed because of the pandemic, and how it should be organized in the future. Excerpts from the German interviews have been translated into English for the reader’s convenience. For the purpose of anonymization, the numbering of the interviewees does not correspond to the order of the interviewees as depicted in Table 2.

**Figure 2:**
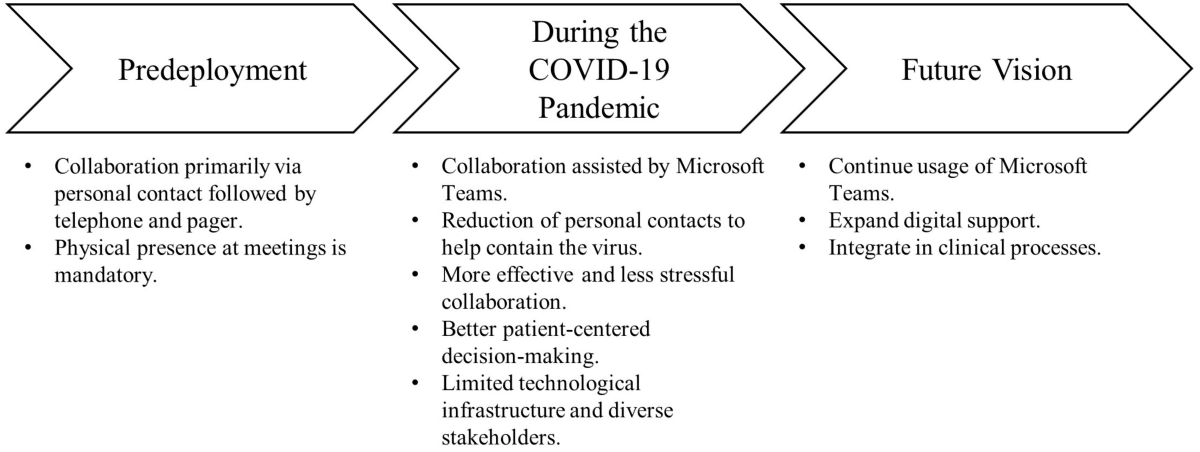
Summary of case study results.

Figure 2 summarizes the results.

### Predeployment Conditions

In every clinic in the hospital, an early meeting is held in which all physicians meet to discuss patients collaboratively and to derive diagnoses and therapies (eg, E1, E2, and E5). Physicians from other disciplines frequently participate by offering their medical expertise. Afterward, physicians conduct ward rounds, perform surgeries, or treat patients. In many disciplines, additional meetings are held in the afternoon to discuss treatment procedures for specific patients:

It depends a bit on the department, but it is generally discussed who is assigned where, the night shift is discussed, X-rays are viewed together with a radiologist, at noon there is another meeting in which current cases are discussed with the senior and chief physician.

E5

Collaboration between physicians in hospitals is characterized by face-to-face or telephone communication (eg, E4, E10, E12, E14). In many cases, personal communication is conducted on the same hierarchical level and within departmental boundaries. For example, information exchange among resident physicians takes place in person, while senior physicians or head physicians are usually approached via telephone:

Much usually happens verbally, with colleagues from the same level.

E3

Most personal communication with other resident physicians is according to our schedule. If I need to talk to a senior physician, I usually call him [or her].

E2

However, not every physician has their own phone; some have pagers (eg, E1, E6). If it is necessary to contact a physician, the number of their pager is called from any telephone in the clinic. The pager will then display the number of the caller with a request for a callback. The physician must find a telephone to respond to the call. Up to this point, the identity of the caller and the reason for the call remain uncertain. In our study, the physicians explained that no official IT system facilitates collaboration (eg, E1, E5, E12). It was also described that existing IT systems lack ease of use and are rather difficult to handle. Furthermore, the velocity of the current infrastructure needs improvement, and retrieving information is not straightforward because medical data are stored across systems (eg, E3, E6, E12):

In the rarest cases communication is done by email, but only when I am sitting in my office. There is no use of any IT system; we are rather old-school.

E6

Systems are frequently just slow. Handling is not intuitive and programs crash.

E3

The physicians reported that communication and collaboration in the hospital are sufficient but are dependent on collaborators adhering to certain organizational structures and communication channels (eg, E1, E7). Problems occasionally arise, such as misunderstandings in shift plans; colleagues can be difficult or impossible to reach, such as when a physician is in the operating theatre; or a faulty transfer from aftercare to day care can be made when information is captured in handwriting (eg, E2, E8, E9):

There are problems when changing shifts if a list has only been filled out by hand and you do not see each other.

E8

However, the physicians explained that no serious issues arise during habitual collaboration (eg, E9, E13, E14) but likewise admitted that the current mode of communication is not being questioned and certainly could benefit from adjustments (eg, E1, E11, E12).

### Collaboration During the COVID-19 Pandemic

Prior to the pandemic, Microsoft Teams was not used in the clinic, nor did any other standardized system exist for collaboration between clinicians in the hospital.

The technological support for the collaboration was equally accepted, but some behaviors changed. Due to the reduction of personal contact between clinicians, the communication was more professional and less social (eg, E2, E6, E10, E14, E16). Communication over the telephone decreased significantly, and many issues were resolved directly via chat. In addition, response times were shorter, and information was shared digitally (eg, E3, E8, E10, E14):

It is already a somewhat different culture which is much more impersonal. There is less personal interaction.

E5

The experts saw various advantages in technical assistance for collaboration. Foremost was compliance with the current rules and hygiene regulations (eg, E1-E6). Collaboration between physicians became easier. Data could be shared directly with everyone, and information was immediately captured digitally. Physicians could access data from anywhere, and even a physician who had not attended a meeting could obtain information via digital protocols. Colleagues could be reached much more quickly, and interdepartmental communication was simplified (eg, E5, E9, E12):

My colleagues are easier to reach, there is a closer connection between us. Medical information is available for everyone and decisions can be made faster and better.

E8

In addition, the IT system simplified group meetings. The experts often complained that in large meeting rooms, the presentation screen was simply too far away (eg, E1, E2, E16). Relevant information was more visible; for example, patient x-rays could be interpreted more easily. Resident physicians reported a learning effect from the joint discussions of pictures (eg, E9, E13, E16):

When you look at the pictures, you get more opinions, they can be used as a basis for a decision, someone can also intervene, if you can see the pictures well, there is also a learning effect.

E4

Meeting via Microsoft Teams was also perceived by the physicians as less stressful and more efficient (eg, E5, E7). Information could be looked up quickly during an appointment. Subsequent discussions could be kept within limits, and there was no longer a need to wait for participants on their way to a meeting room (eg, E8, E13). In addition, there was no need to use unknown hardware from an unfamiliar room for a presentation. Furthermore, experts reported that one could better prepare for transferring patient information:

As a physician, you can prepare yourself better, the information is already there. Everyone can prepare themselves and you don't have to do it together.

E4

In addition, the physicians recognized indirect advantages for patients. The more effective and efficient appointments saved time, which could be used for more relevant tasks such as diagnosis or planning treatment. Through the joint discussion of patient reports, several professional opinions could be obtained, possibly leading to better decision-making (eg, E6, E12, E14). Finally, in the time of the pandemic, the potential risk of infection was minimized:

I believe we physicians are the greatest threat to everyone.

E9

This new type of collaboration requires cognitive effort and a high level of self-discipline. Physicians have a highly interactive job. During a pandemic, they are not always allowed to meet a colleague and must encourage themselves to participate in digital communication. There is a feeling of a need to be constantly available, which creates stress and the perception of being controlled (eg, E4, E8, E9). A physician is always expected to be up to date, even when working from home or when on vacation:

But it is expected that you always look in and up to date, even if you are at home you still look in there, you are afraid to miss something out.

E7

Finally, challenges of the collaborative adjustment were described. In addition to the partially slow internet connection, the new hardware had to be purchased quickly. Very few computers were equipped with proper cameras, microphones, or loudspeakers; therefore, collaboration with colleagues was more difficult (eg, E1, E2, E12):

Everybody had to install the software by himself and hardware was only available little by little. I couldn't log in from the office. The internet connection was bad so that people couldn't understand each other.

E2

The system had many diverse users, such as resident and senior physicians. The system was used with varying degrees of intensity and not to its full extent. The full range of functions of the software was not manageable from the beginning, and the users were required to learn the functions themselves (eg, E2, E7, E12):

Everyone used it the way they thought was right. We just got it.

E11

### A Vision of the Future

In the last part of the interviews, we asked the physicians how a future collaboration supported by technology would look. Some stated that Microsoft Teams should continue to be used, as it is broadly accepted and adopted (eg, E1, E2, E7). In addition, the change from classical messaging apps such as WhatsApp toward Microsoft Teams for professional purposes was already visible (eg, E7, E8):

Organizational things are shared different. It just works better.

E7

However, the different types and levels of use must be considered. On the one hand, the technological support for collaboration should be further integrated. For example, physicians tend to continue virtual meetings in the future, as those are particularly helpful when individuals from different clinical disciplines need to participate and the physical attendance of participants is not required (eg, E3, E4, E11). In addition, information exchange among physicians is likely to change, as medical data are easier to retrieve (eg, E6, E13):

I would like to keep the tumor board meetings going like this. Everyone sits at their own workplace; x-ray images are easier to see and disease patterns better to recognize; ad-hoc information for a specific case can be obtained quickly.

E5

I think we should continue to use this especially for appointments that are attended by many people from different disciplines or in the future for such things as e-learning on surgery techniques.

E3

There are some useful areas where we can use [Microsoft] Teams. A lot of our digital communication is conducted via email. I think this might be limitable or even replaceable for internal information exchange.

E6

On the other hand, personal contact is still fruitful and frequently the more favored means of collaboration. Especially for younger physicians, who are at the beginning of their medical training and rather inexperienced, personal contact is indispensable for conveying medical knowledge and demonstrating practical techniques on patients (eg, E1, E15, E16). Furthermore, mere virtual communication may result in negative consequences for teamwork and team spirit in the hospital (eg, E2, E5, E13):

When someone explains something to me, it’s easier to understand when we meet in person. I can't imagine how virtual meetings look like when treating or operating patients where it is about the practical execution.

E15

We spend so much time together, I sometimes want personal interaction with my colleagues. Unfortunately, this is currently somewhat lost.

E13

I think [virtual collaboration] is especially beneficial when you already know each other and each other’s work.

E5

Therefore, not every type of collaboration should be supported or replaced by technology. However, the physicians explained that the current pandemic is forcing hospitals to undertake collaborative alignments (eg, E4-E8) as necessary endeavors to limit personal contact to a minimum, some of which should have been undertaken even sooner (eg, E4, E9):

We should continue this kind of virtual collaboration. I think we need to cope with the restrictions [due to the pandemic] for some time.

E10

We need the implementation as quickly as possible during a pandemic.

E7

Finally, technology may improve not only collaborations among physicians (and other clinicians) but those with external partners. For example, some physicians reported that scientific collaborations have been initiated for research purposes (eg, E1, E2) with participants across departmental boundaries, that is, from internal clinics, external laboratories, and independent institutions.

## Discussion

In this section, we present the most relevant insights gained from our case study. We explain general key lessons learned and offer recommendations on how digital transformation can improve collaboration. Our goal is to guide hospitals and their decision makers who want to embrace digital transformation for collaborative purposes.

### Lesson 1: Organizational Change Requires Alignment of the Mindset

Digital transformation can change the way clinicians collaborate in hospitals. Achieving organizational change is not easy in surroundings that are characterized by hierarchical and authoritarian individuals [36]. IT-enabled transformation does not always proceed as desired [40], as working practices in hospitals have not greatly changed in the last few decades. Although the technological possibilities have matured and investments continue to rise [64], collaboration is still conducted with common, proven instruments [65].

In our study, the physicians’ experience was that the assistance of an IT system simplified tasks in many areas of their daily working life. Simplification enhanced adaptations in the types of collaboration, moving from personal contact to a virtual environment. Aligning one’s mindset required a high degree of self-control to avoid falling back into habitual patterns. One expert described this as “a different kind of communication that requires effort and self-discipline in a profession that is strongly characterized by personal interaction” [E5]. However, physicians observed that this alignment is necessary because “otherwise everyone wants to do it like we did before” [E2].

Recommendation: Hospitals should develop an overarching adoption strategy to meet the varying expectations of involved stakeholders. The introduction of technology today differs from that of 20 years ago. IT is simply a means to an end, and it should not be the center of attention. Instead, it provides a new way of working and of transforming certain processes. The entrenched mindset can only be changed piece by piece, accompanied by a variety of formats. This deliberate process might include different training sessions or workshops, such as how clinicians can collaborate to jointly develop an IT system to support, improve, or even completely replace certain processes.

### Lesson 2: Develop and Adhere to Behavioral Rules for Collaboration

Enabling new forms of collaborative work drives operational performance, not only within the clinic but across departmental boundaries [30,31]. The introduction of IT systems in hospitals is transformative and may be disruptive [66]. Furthermore, technological capabilities are broadly available to improve information exchange and collaboration [45-47]. The existence of technology does not necessarily describe how to handle that technology. Systems may be used in various ways; however, there is no “right” or “wrong” when it comes to the individual handling of a system.

In our work, physicians described a system that is used for different purposes with various functions. Some clinicians conducted video calls, while others used the chat function or shared files. Many questions remained unanswered: Which functions should be used at all? How quickly should one react to chat messages? Is a reaction also expected outside regular working hours? Which files are shared, and in what type of structure are they stored? Questions should be clarified in advance to ensure a structured collaboration. One expert explained that “there were communication problems, cameras were bought without anyone knowing. We need rules on how to act and how to behave” [E2].

Recommendation: Before or during the introduction of new technology, each clinic should define rules for its behavior and IT-enabled collaboration with stakeholders. Changes can never be initiated and sustained alone. The goal of improved collaboration and thus enhanced patient care can only be achieved if everyone participates and follows certain rules. The change should be embraced collaboratively.

### Lesson 3: Antiquated Technical Infrastructure Hinders a Shift

Achieving cross-functional collaboration among hospital physicians presupposes the integration of suitable technologies to improve the overall treatment of patients [27,28]. To enhance the overall diagnostic and treatment process further requires IT systems with stored information to be accurate, relevant and integrated as well as quickly accessible when needed [35,36]. However, hospitals still struggle to adopt new technologies in their complex clinical environments and to integrate them into their underlying infrastructure [52].

Our results revealed that physicians appreciated the IT-enabled support and wanted to continue to use it; however, they felt restricted due to technical problems. The participants reported that the internet connection in the hospital regularly failed or was only adequate for transmitting sound without a video signal. In addition, the computers were all stationary and frequently outdated. The lack of devices, in turn, led to many physicians using private devices, on which data protection and security were problematic. Physicians simply observed that “the infrastructure must be available” [E1] and “the technology must be upgraded and ideally interlinked in all parts of the health care system” [E4].

Recommendation: Hospitals must provide technical fundamentals to promote IT-enabled collaboration between physicians. This training applies not only to the infrastructure itself, such as network availability, but to the technical equipment. Every physician could be equipped with appropriate devices such as notebooks, tablets, or smartphones. A positive outcome of this would be that the equipment managed by the clinic’s IT department would comply with data protection regulations.

### Conclusions

Digital transformation, with all of its disruptive approaches, improves collaboration between clinicians, not only during crises such as the COVID-19 pandemic. Hospitals are slowly turning into smart hospitals that incorporate innovative technologies with optimized and automated processes to provide better patient care [42-44]. Technical advancements had been available before [45-47]; however, a crisis forced the introduction of IT-enabled assistance [55]. The reduction of personal contact among physicians to contain the virus and lower the infection rate was the ultimate objective.

In our study, physicians positively reviewed the introduction of the IT system to enhance collaboration in their hospital. However, it is necessary to ensure that the technical basis for the use of the system is available. Furthermore, it takes time for an organizational change to be embraced. Finally, rules for virtual collaboration should be defined and should be followed by every stakeholder.

The rapid and unavoidable introduction of IT-enabled collaboration revealed profound grievances concerning the technological requirements of hospitals and the digital expertise of clinicians. Digital dissemination of medical information along the patient treatment path is not entirely complete. Medical data are exchanged verbally or in handwriting and are often not available throughout the hospital [15,22]. Technology has found its way into our everyday lives, but physicians are still not fully aware of the possibilities that digital transformation reveals [65]. There is a lack of practice and actual use within hospitals, as IT-enabled collaboration is not necessarily needed, and the health care sector traditionally lags in adopting new technologies [30,67].

To avoid being caught unprepared by future crises, digital transformation must be further driven to ensure effective and efficient collaboration even without personal contact. Such virtual collaboration requires the diagnostic process and subsequent treatment to be opened to disruptive strategies. Openness will not only contribute to faster change but will embrace patient-oriented behavior. Moreover, a rapid rollout contributes to quicker adoption. The adoption of pragmatic and innovative solutions will increase their acceptance for future use.

## Abbreviations

AI: artificial intelligence
CSCW: computer-supported cooperative work
EHR: electronic health record
HIT: health information technology
IoT: Internet of Things
IT: information technology
